# Supplementary figures and images for: The shapes of elongating gastruloids are consistent with convergent extension driven by a combination of active cell crawling and differential adhesion
Source: PLoS Comput Biol. 2024 Feb 2;20(2):e1011825. doi: 10.1371/journal.pcbi.1011825 (PMC10866519; doi:10.1371/journal.pcbi.1011825)

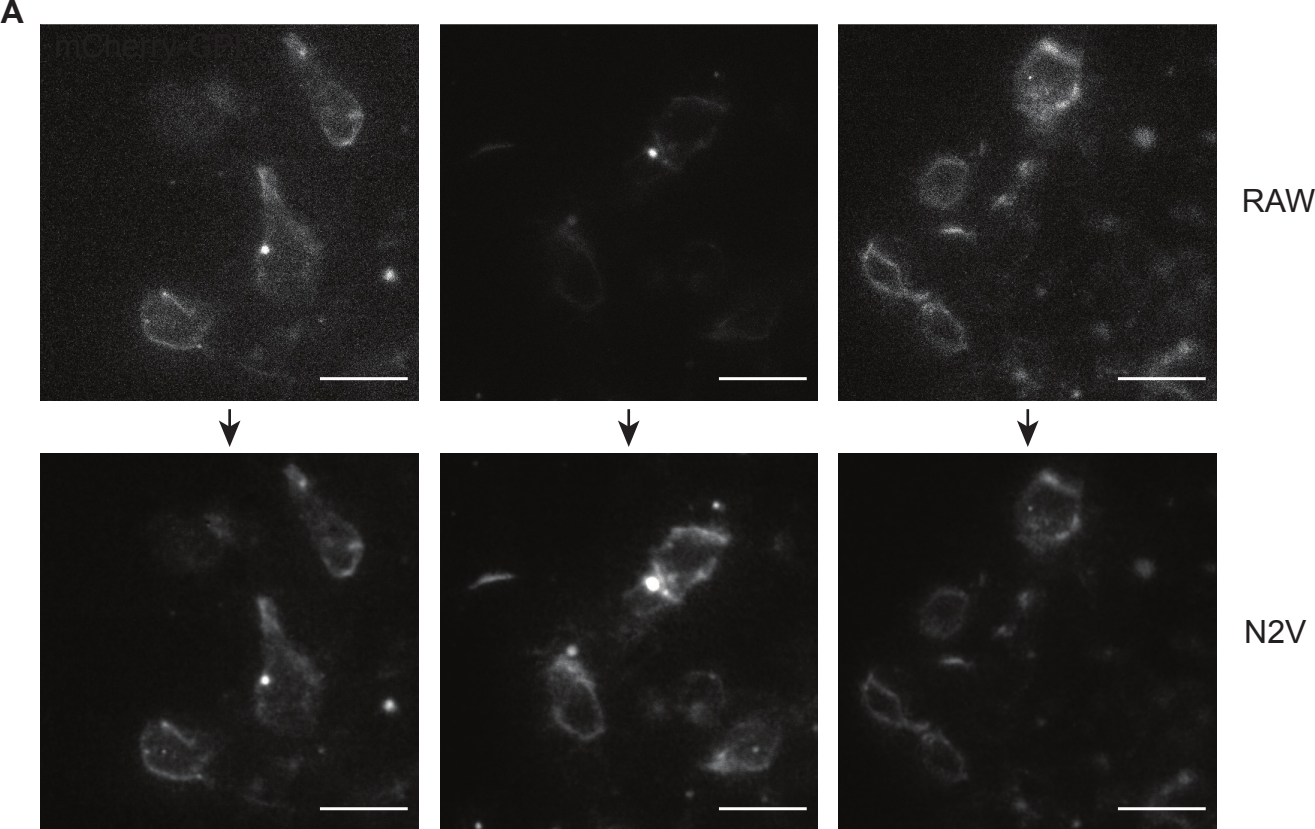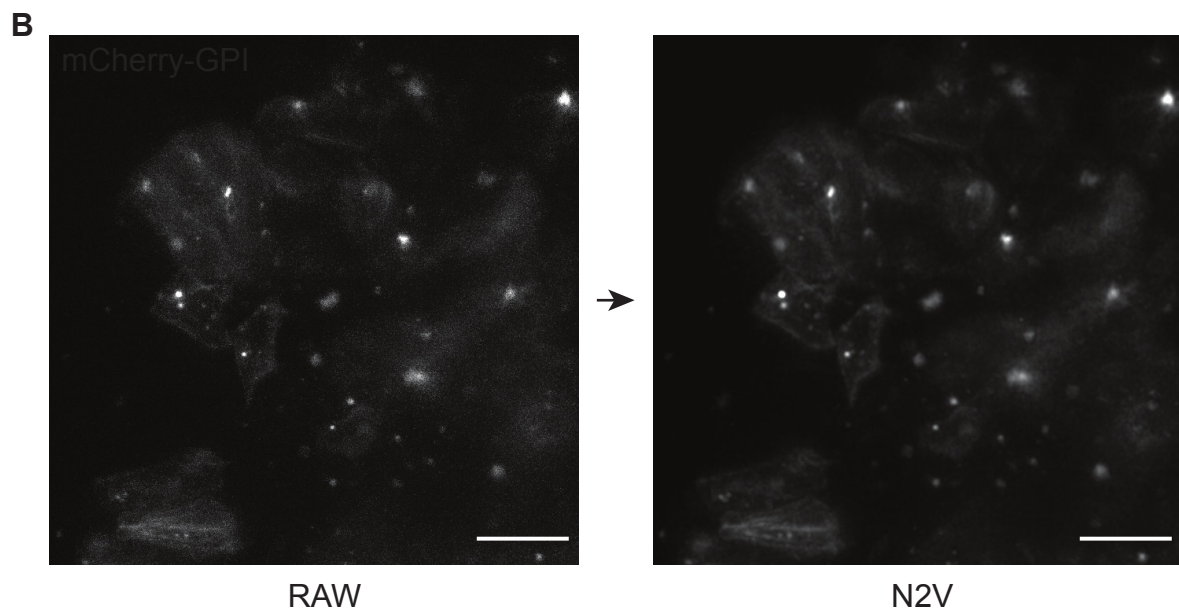

Supplement: S1 Fig — A: Image of raw data (top panel) and denoised data (bottom panel) at t = 1. A cropped image (505 × 505 pixels) is shown of the 11th plane of the z-stack of the 1st time point of the time lapse. The denoised image was predicted from the model that was trained on the images of the 1st time point. B: Image of raw data (top panel) and denoised data (bottom panel) at t = 5. A cropped image (505 × 505 pixels) is shown of the 10th plane of the z-stack of the 5th time point of the time lapse. The denoised image was predicted from the model that was trained on the images of the 7th time point. C: Image of raw data (top panel) and denoised data (bottom panel) at t = 23. A cropped image (505 × 505 pixels) is shown of the 6th plane of the z-stack of the 23rd time point of the time lapse. The denoised image was predicted from the model that was trained on the images of the 31st time point, the last time point of the time lapse. D: Image of raw data (left panel) and denoised data (right panel) at t = 31. A cropped image (674 × 674 pixels) is shown of the maximum projection of the z-stack of the 31st time point of the time lapse. The denoised image was predicted from the model that was trained on the images of the 31st time point. A,B,C,D: The minimum displayed value for each image was set to the 5th percentile of the image intensity distribution to enable a qualitative comparison. Scale bars: 20 μm. (PDF) [file pcbi.1011825.s001.pdf]

**A**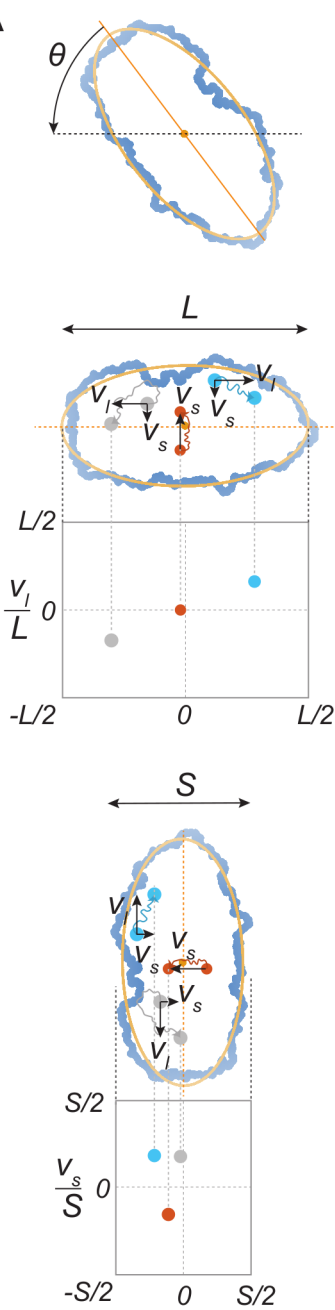**B**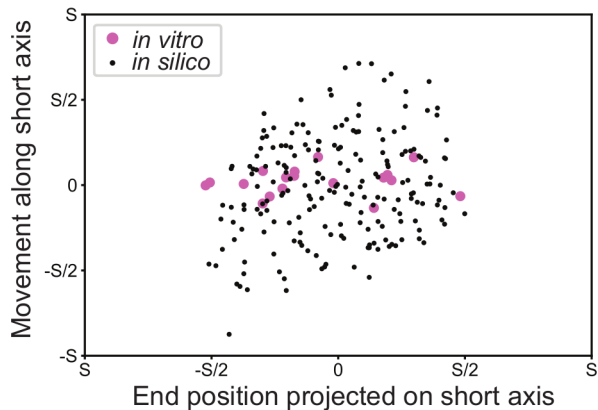**C**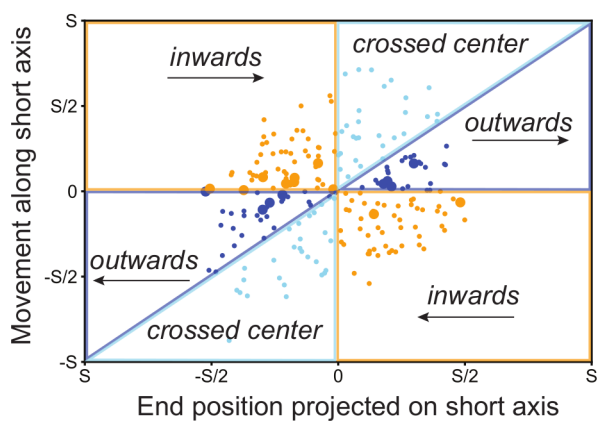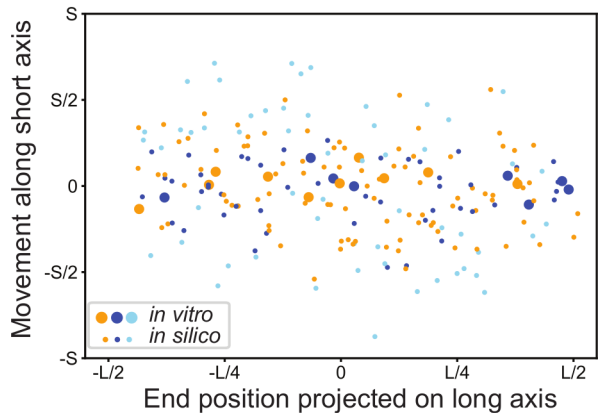

Supplement: S2 Fig — A: Scheme showing how cell positions were projected on the long axis of the gastruloid. An ellipse was fitted to the outline of the gastruloid. The angle θ between the long axis of the ellipse and the nearest orthogonal axis when rotating counter-clockwise was determined for each time point (top panel). Positions of cells were rotated by the angle θ to project them on the long axis of the gastruloid. Here, the new x coordinate of each cell corresponds to the cell’s position on the long axis, and the new y coordinate to the cell’s position on the short axis. For each cell, the movement along the long axis vl (xend − xstart) or along the short axis vs (yend − ystart) was determined, and normalized by the length of the long axis L or the short axis S, respectively. The resulting vl/L was plotted against the end position of each cell projected on the long axis (mid panel). The resulting vs/S was plotted against the end position of each cell projected on the short axis (bottom panel). B: Distance each cell has moved along the short axis plotted against the end position of each cell projected on the short axis. Large, pink circles represent measured cells. Small, black circles represent cells in the simulated gastruloid. C, top panel: Same plot as in B with data points colored by the direction of movement with respect to the short axis. Orange: cells are moving inwards, towards the long axis; dark blue: cells are moving outwards, away from the long axis; light blue: cells that cross the long axis, thus first move inwards, then outwards. Bottom panel: distance each cell has moved along the short axis plotted against the end position of each cell projected on the long axis. Cells are colored in the same way as in the top panel. Large circles represent measured cells. Small circles represent cells in the simulated gastruloid. (PDF) [file pcbi.1011825.s002.pdf]

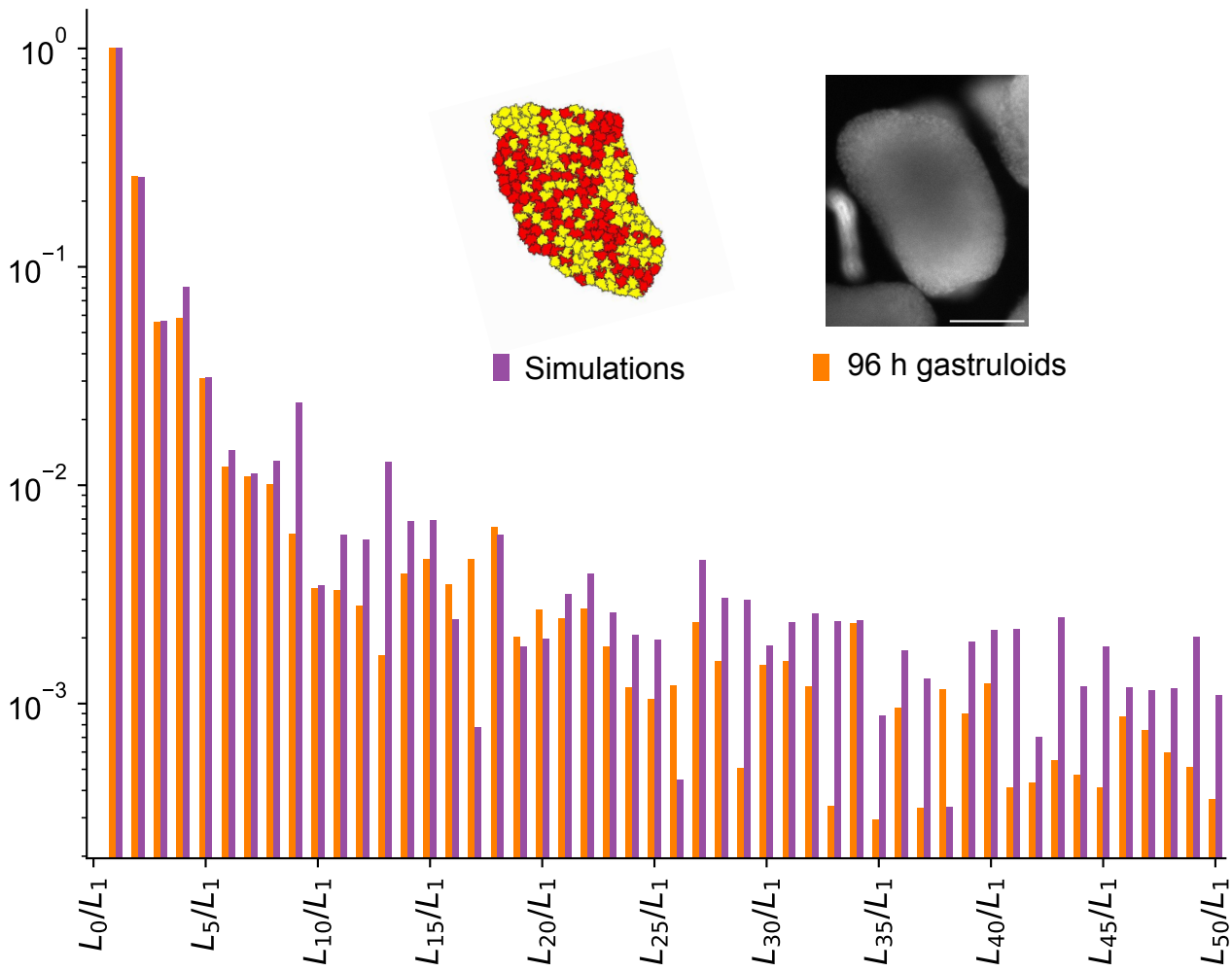

Supplement: S3 Fig — Similar measured and simulated gastruloid shapes are compared (see also Fig 4E). Lattice effects and simulation noise result in bigger contributions of higher modes in the simulated shape compared to the measured gastruloid. (PDF) [file pcbi.1011825.s003.pdf]

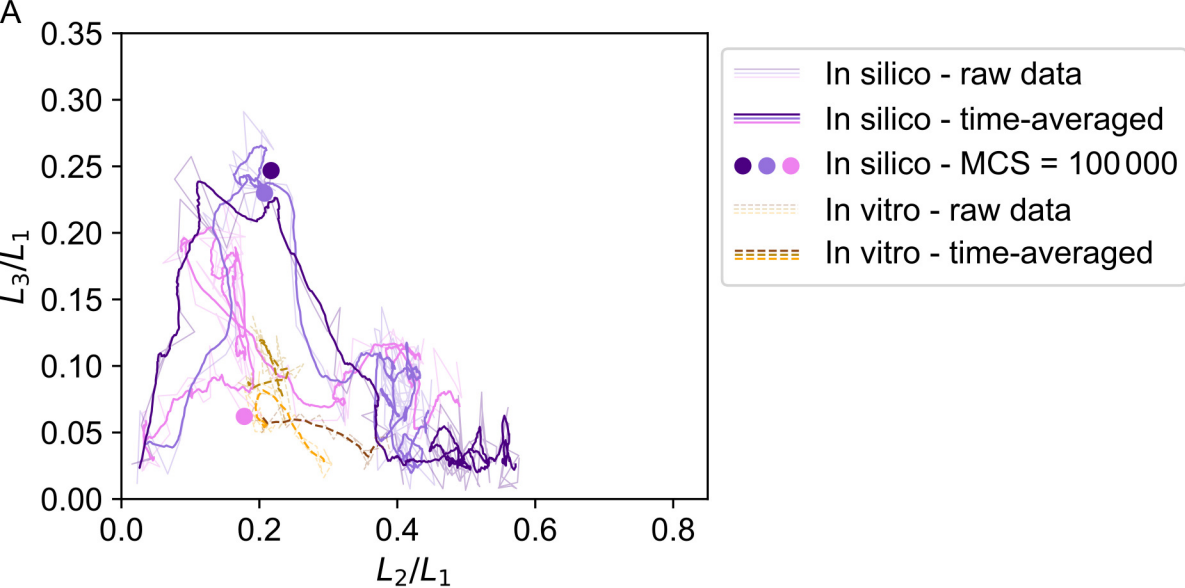

One cell type

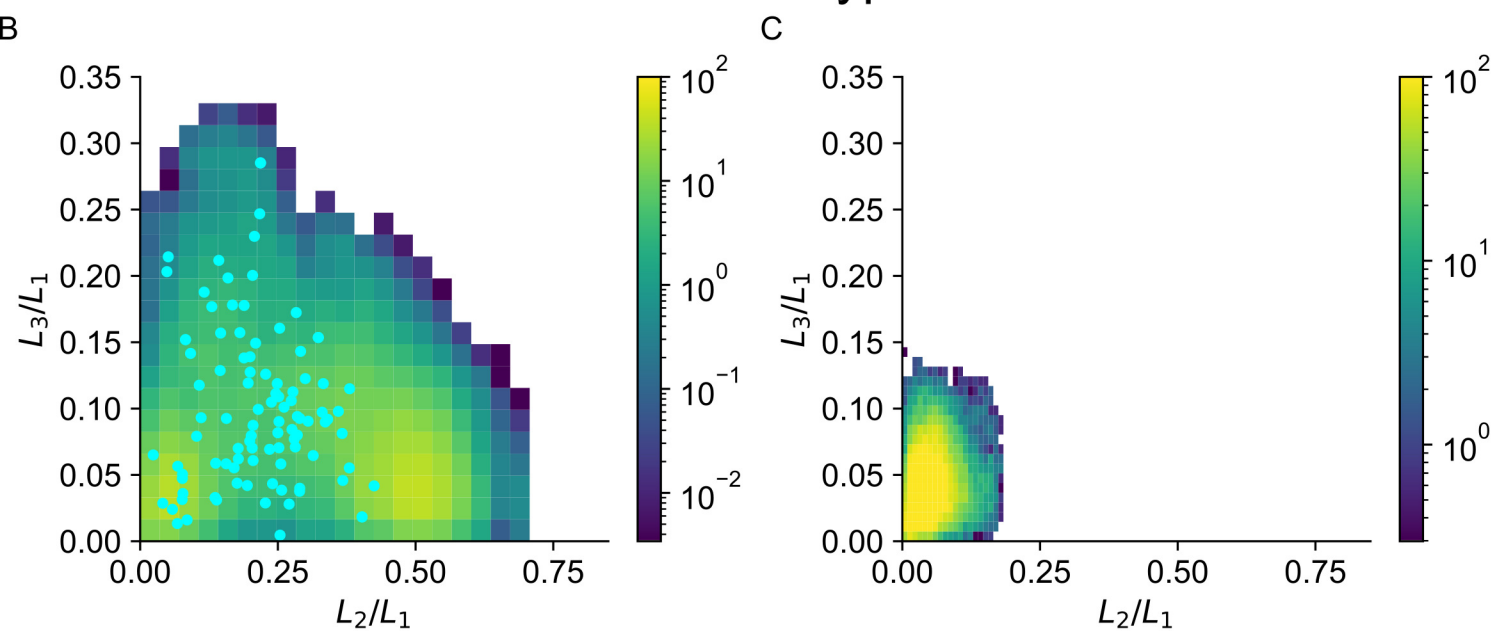

Two cell types

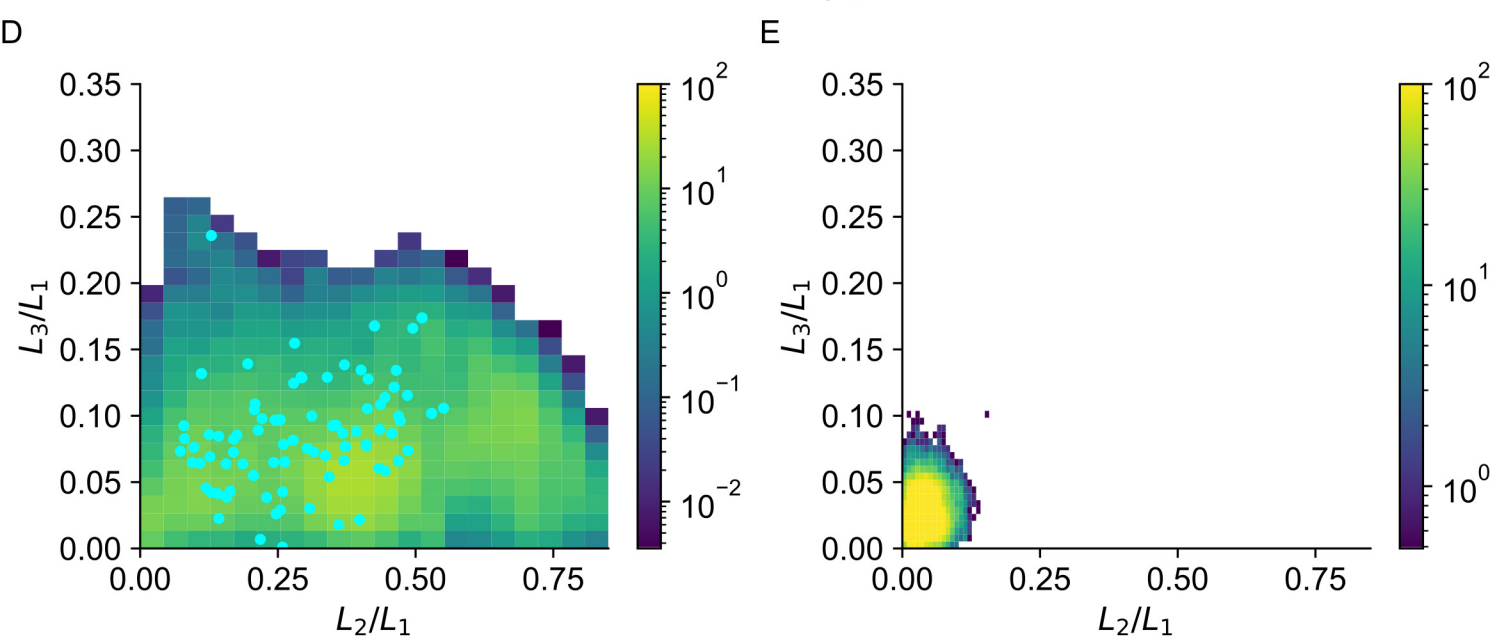

Supplement: S4 Fig — A: The evolution of three simulations with the same parameters (pulling force λF = 20 and γ(c, M) = 15) and three in vitro time lapses in the L2/L1 − L3/L1 space. The simulations are performed for a prolonged time of 500,000 Monte Carlo steps. The thin solid lines show the L2/L1 and L3/L1 values every 5,000 Monte Carlo steps. The thick solid lines indicate values averaged over 25 measurements (each 500 Monte Carlo steps apart). The dots highlight the values at 100,000 Monte Carlo steps, which is the simulation time in all other figures. The dashed lines represent the shapes of gastruloids measured by in vitro time-lapse microscopy from 72 h to 94 h, 91 h 20 m and 88 h 40 m respectively for the orange, golden and brown curves. The gastruloid shapes were obtained manually. The thin lines show the raw data (each 40 minutes apart), and the thick lines show time averages over 200 minutes. B: Distribution of the L2/L1 and L3/L1 values for 100 simulations with the same parameters at different time points, up to 500,000 Monte Carlo steps. The values at 100,000 Monte Carlo steps are indicated with dots. C: Distribution of the L2/L1 and L3/L1 values for 100 simulations with the same parameters at different time points without pulling, up to 50,000 Monte Carlo steps. D: Same as B, but with two cell types and parameters γ(1, 2) = −4 and λF = 20 as in (Fig 4D). E: Same as C, but with two cell types and parameter γ(1, 2) = −4. (PDF) [file pcbi.1011825.s004.pdf]

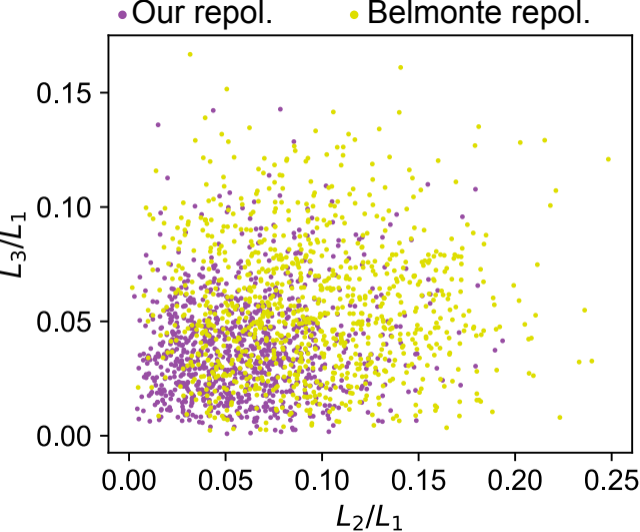

Supplement: S5 Fig — A 2D Kolmogorov-Smirnov test comparing the shape distributions yielded D = 0.41 and p = 7.4 ⋅ 10−30. (PDF) [file pcbi.1011825.s005.pdf]

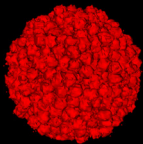

1000 MCS

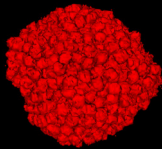

20,000 MCS

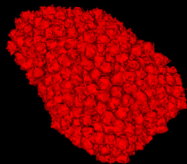

40,000 MCS

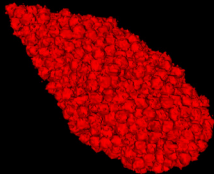

60,000 MCS

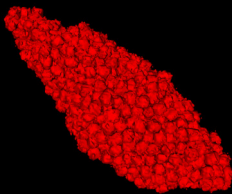

80,000 MCS

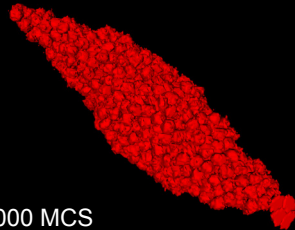

100,000 MCS

Supplement: S6 Fig — The same parameters as the optimal parameters in (Fig 4) were used, except for a pulling force of λF = 150. For 100,000 MCS, cells came in contact with the simulation boundary, see S1 Video. (PDF) [file pcbi.1011825.s006.pdf]

# A "All-all" pulling

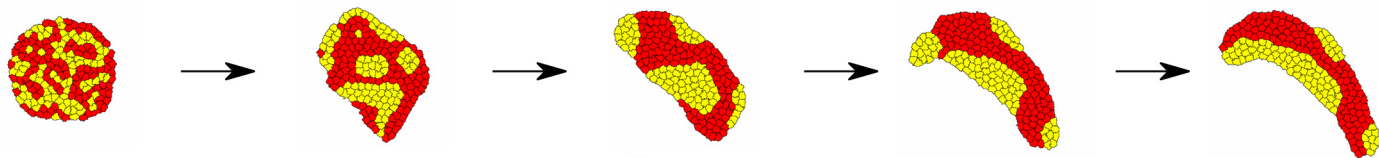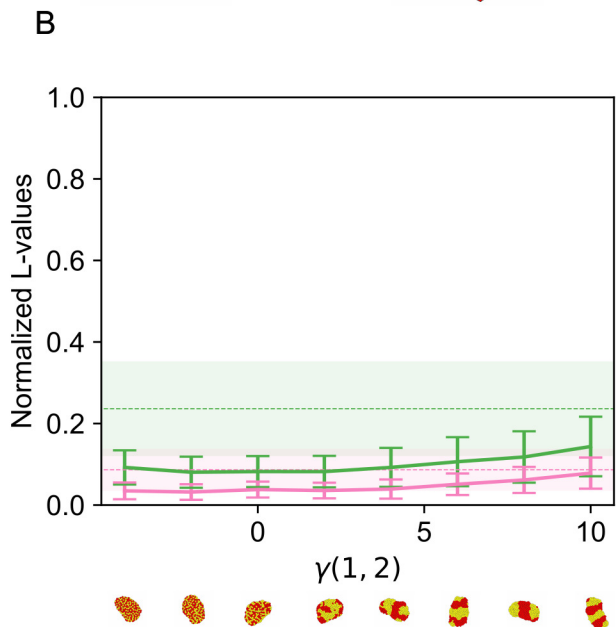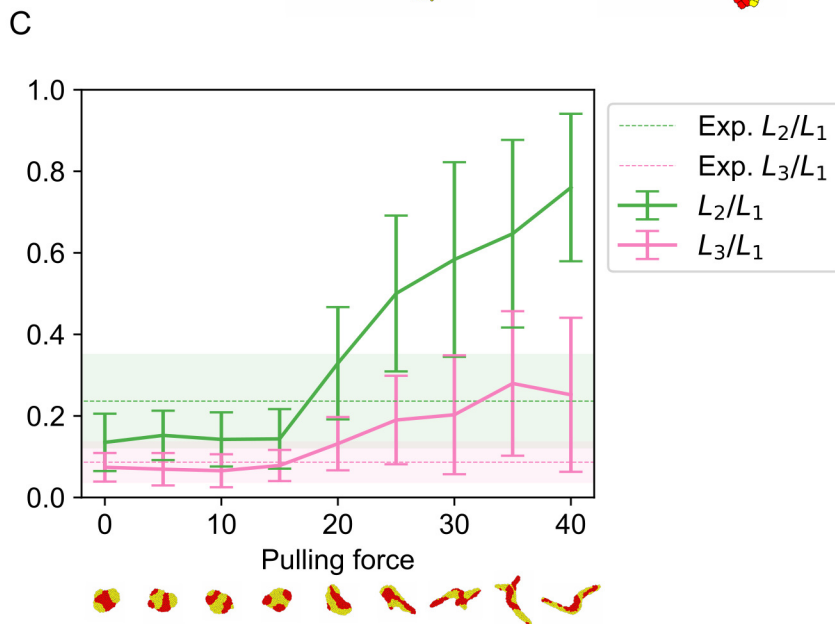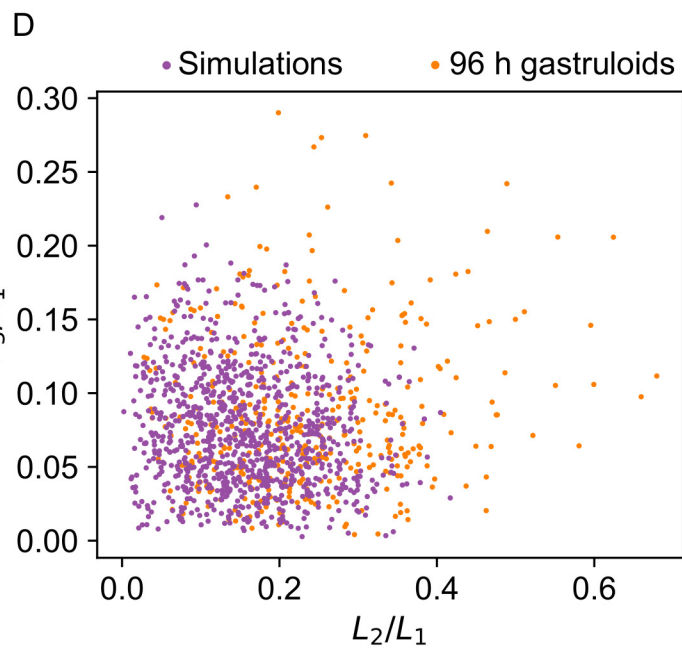

**E**

$L_2 / L_1 = 0.226$   
 $L_3 / L_1 = 0.120$

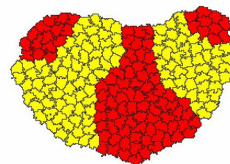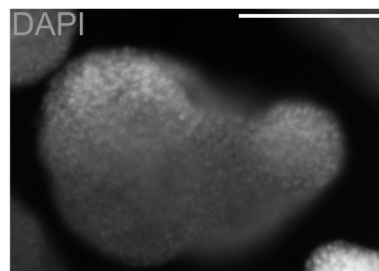

$L_2 / L_1 = 0.226$   
 $L_3 / L_1 = 0.119$

Supplement: S7 Fig — A: Example shapes resulting from a simulation of all-all pulling for 100, 25,000, 50,000, 75,000 and 100,000 Monte Carlo steps. B,C: Quantification of shapes resulting from simulations of CE driven by crawling and differential adhesion with two cell types. Shown are LOCO-EFA coefficients averaged over a population of shapes. The shaded areas (in the experimental data) and vertical bars (in the simulation data) indicate standard deviations. B: The surface tension between the cell types was varied. The pulling force was kept constant at 15. C: The pulling force was varied. The surface tension between the cell types was kept constant at 10. D: Scatter plot of the scaled LOCO-EFA coefficients L3/L1 versus L2/L1 for the experimental data and simulations of crawling-driven CE with pulling force λF = 15 and surface tension γ(1, 2) = 10. These parameters gave the smallest 2D Kolmogorov-Smirnov test statistic D = 0.37 and p = 1.4 ⋅ 10−7. E: Example of experimentally observed shape (bottom) with highly similar simulated shape (top) and their corresponding scaled LOCO-EFA coefficients L3/L1 and L2/L1. Bottom: a 96 h fixed gastruloid. Shown is the mid-plane of a z-stack. Cell nuclei were stained with DAPI. Scale bar: 200 μm. (PDF) [file pcbi.1011825.s007.pdf]

# A "Yellow-all" pulling

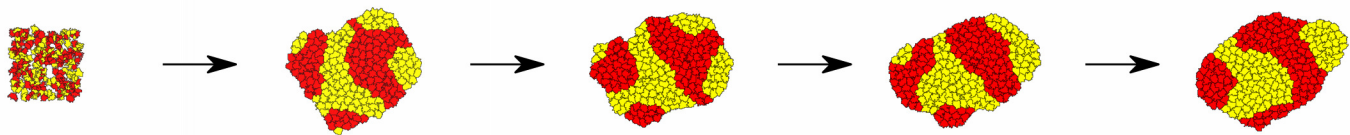

B

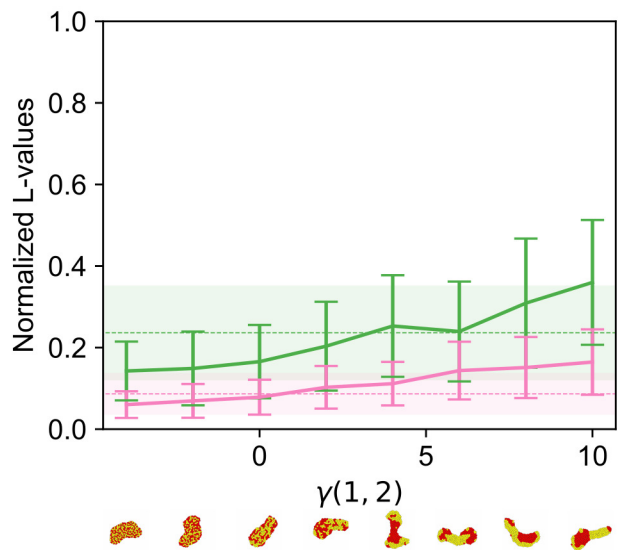

C

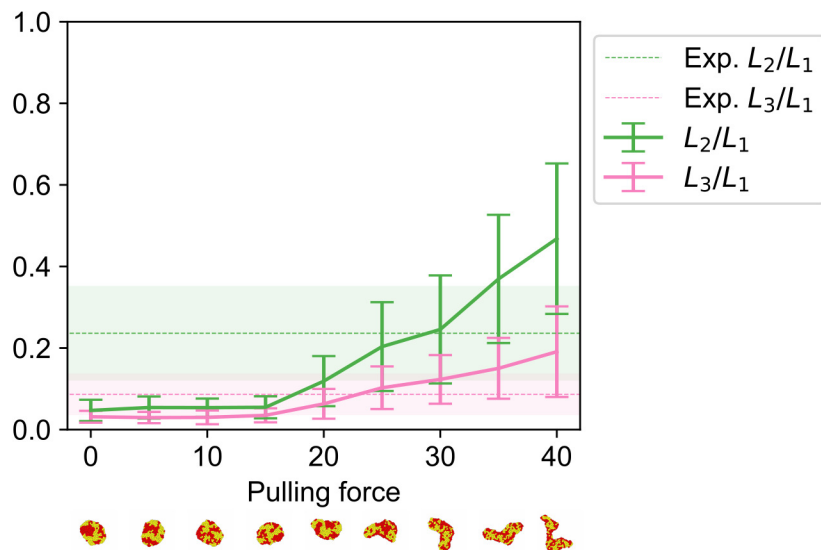

D

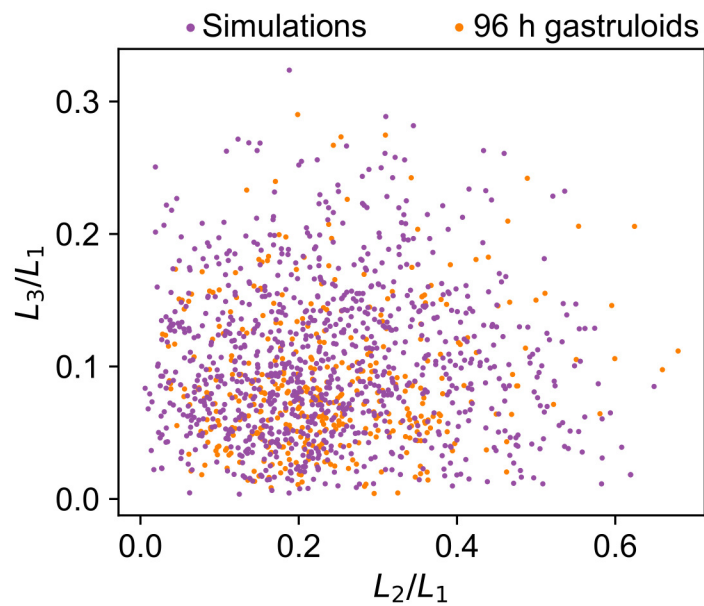

E

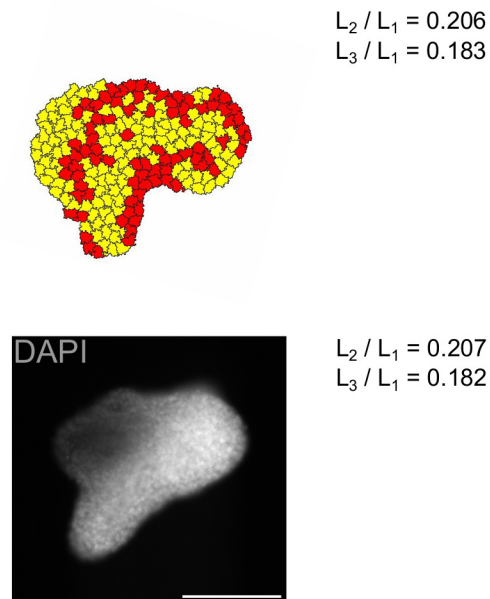

Supplement: S8 Fig — A: Example shapes resulting from a simulation of yellow-all pulling for 100, 25,000, 50,000, 75,000 and 100,000 Monte Carlo steps. B,C: Quantification of shapes resulting from simulations of CE driven by crawling and differential adhesion with two cell types. Shown are LOCO-EFA coefficients averaged over a population of shapes. The shaded areas (in the experimental data) and vertical bars (in the simulation data) indicate standard deviations. B: The surface tension between the cell types was varied. The pulling force was kept constant at 25. C: The pulling force was varied. The surface tension between the cell types was kept constant at 2. D: Scatter plot of the scaled LOCO-EFA coefficients L3/L1 versus L2/L1 for the experimental data and simulations of crawling-driven CE with pulling force λF = 25 and interaction energy γ(1, 2) = 2. These parameters gave the smallest 2D Kolmogorov-Smirnov test statistic D = 0.21 and p = 0.0098. E: Example of experimentally observed shape (bottom) with highly similar simulated shape (top) and their corresponding scaled LOCO-EFA coefficients L3/L1 and L2/L1. Bottom: a 96 h fixed gastruloid. Shown is the mid-plane of a z-stack. Cell nuclei were stained with DAPI. Scalebar: 200 μm. (PDF) [file pcbi.1011825.s008.pdf]

A

"Yellow-yellow" pulling

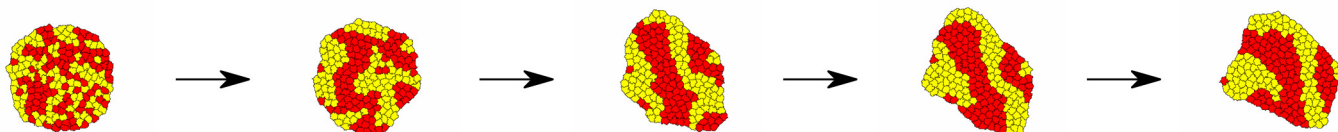

B

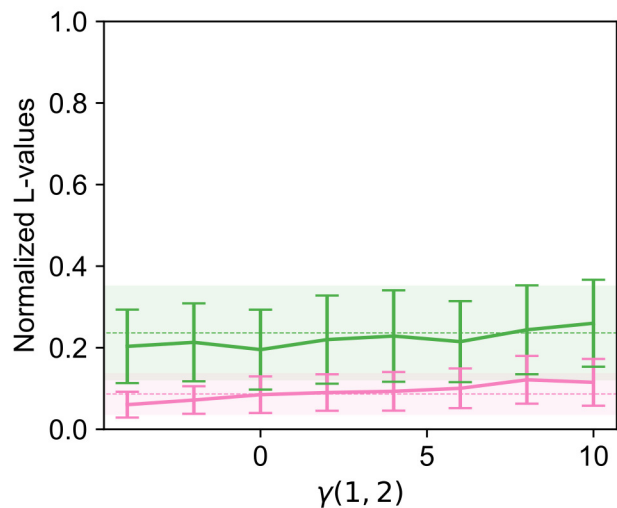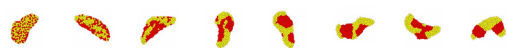

C

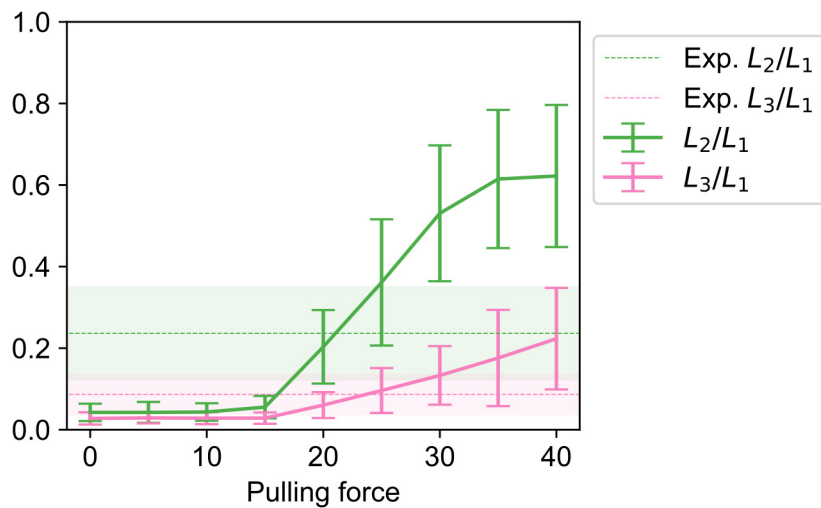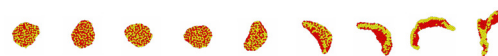

D

• Simulations • 96 h gastruloids

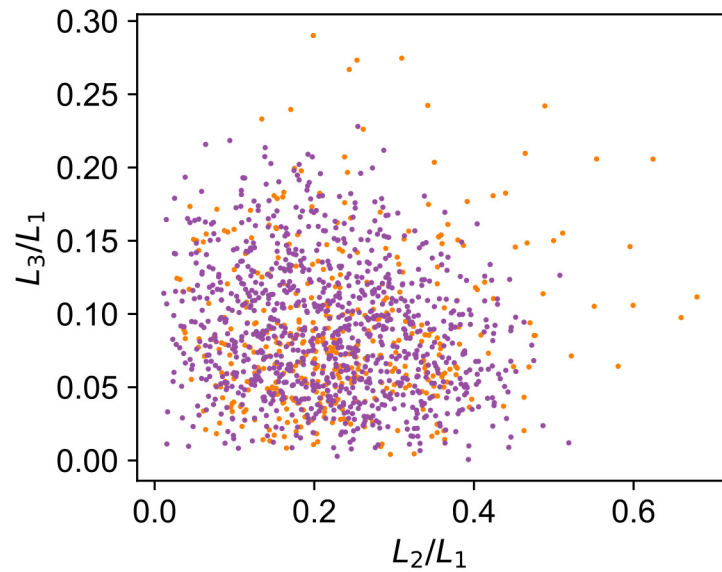

E

$L_2 / L_1 = 0.385$   
 $L_3 / L_1 = 0.149$

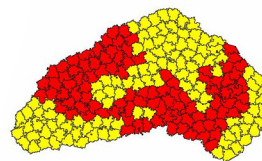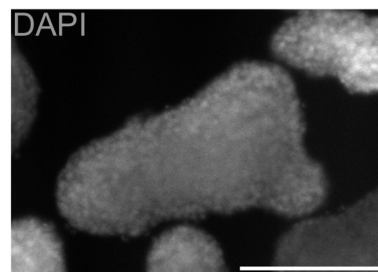

$L_2 / L_1 = 0.380$   
 $L_3 / L_1 = 0.151$

Supplement: S9 Fig — Red cells do not exert or feel filopodia. A: Example shapes resulting from a simulation of yellow-yellow pulling for 100, 25,000, 50,000, 75,000 and 100,000 Monte Carlo steps. B,C: Quantification of shapes resulting from simulations of CE driven by crawling and differential adhesion with two cell types. Shown are LOCO-EFA coefficients averaged over a population of shapes. The shaded areas (in the experimental data) and vertical bars (in the simulation data) indicate standard deviations. B: The surface tension between the cell types was varied. The pulling force was kept constant at 20. C: The surface tension between the cell types was kept constant at 2. D: Scatter plot of the scaled LOCO-EFA coefficients L3/L1 versus L2/L1 for the experimental data and simulations of crawling-driven CE with pulling force λF = 20 and surface tension γ(1, 2) = 2. These parameters gave the smallest 2D Kolmogorov-Smirnov test statistic D = 0.15 and p = 0.12. E: Example of experimentally observed shape (bottom) with highly similar simulated shape (top) and their corresponding scaled LOCO-EFA coefficients L3/L1 and L2/L1. Bottom: a 96 h fixed gastruloid. Shown is the mid-plane of a z-stack. Cell nuclei were stained with DAPI. Scale bar: 200 μm. (PDF) [file pcbi.1011825.s009.pdf]

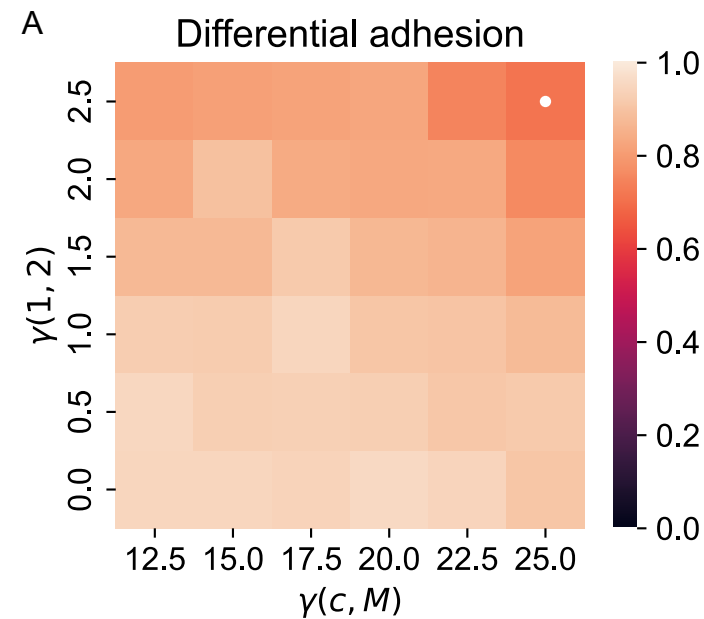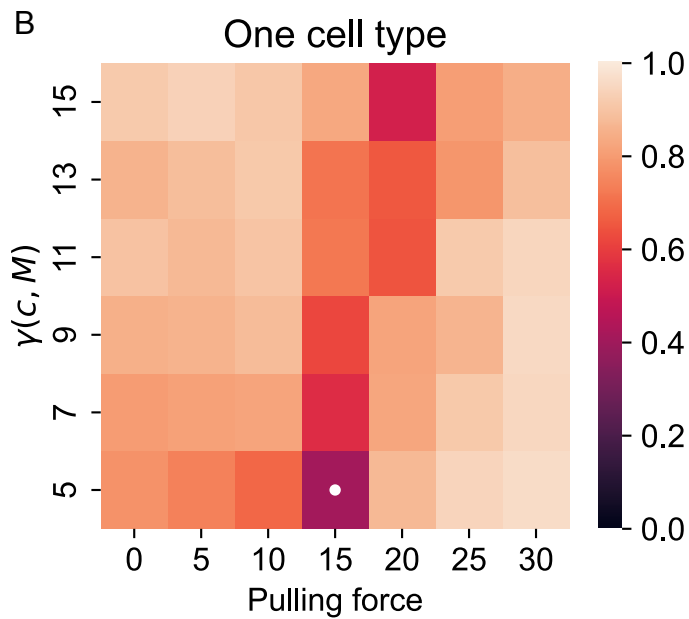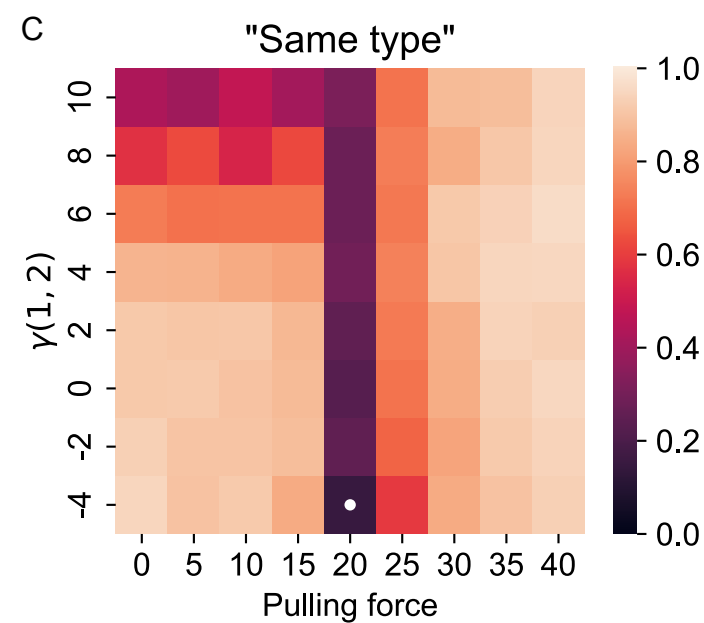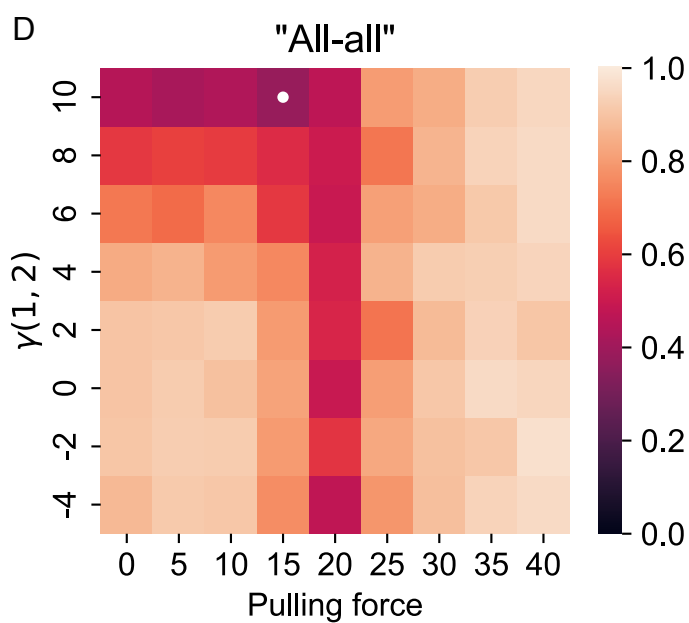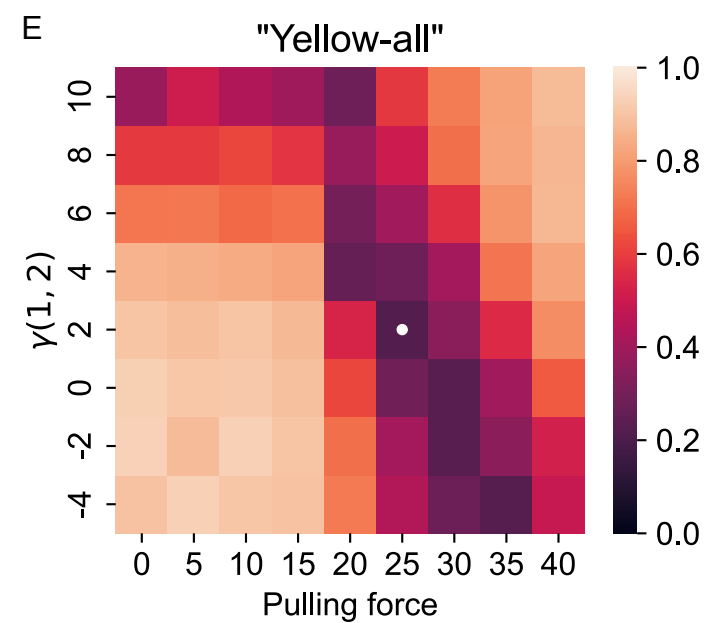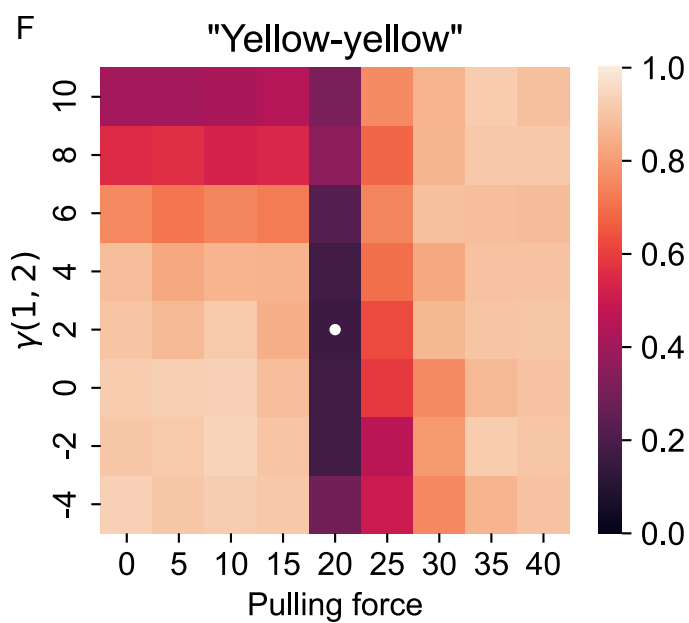

Supplement: S10 Fig — The test statistic D from the 2D Kolmogorov-Smirnov test has been computed from 100 simulated points for different parameter values and different models. The lowest values are indicated with a white dot and represent the best correspondence between the in vitro and in silico shape distributions. A: For differential adhesion. B: For one cell type pulling. C: For same type pulling. D: For all-all pulling. E: For yellow-all pulling. F: For yellow-yellow pulling. (PDF) [file pcbi.1011825.s010.pdf]

A

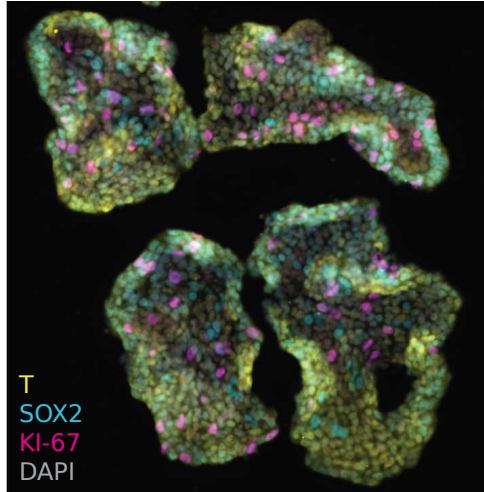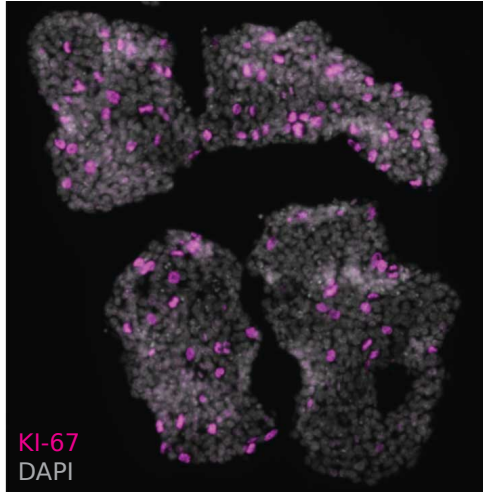

B

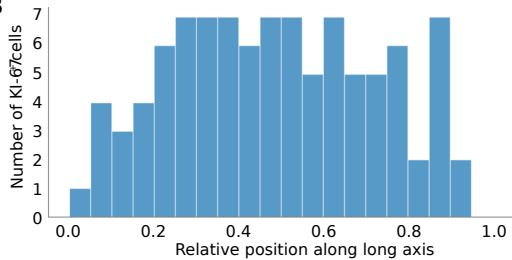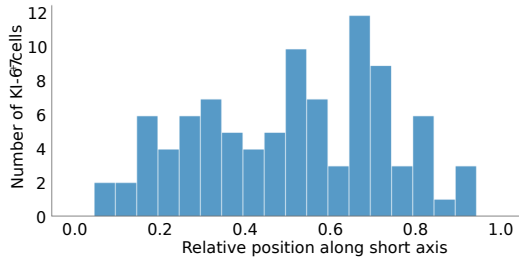

Supplement: S11 Fig — A: Immunostained sections of four gastruloids that were fixed at 96 h. Left: immunostaining of Brachyury/T, Sox2 and the proliferation marker Ki-67. Right: The same sections, but only the immunostaining of Ki-67 is shown. Cell nuclei were stained with DAPI. Scale bars: 100 μm. B: Distribution of the relative positions of proliferating cells (Ki-67-positive) along the long axis (left) or short axis (right) of the respective gastruloid. (PDF) [file pcbi.1011825.s011.pdf]

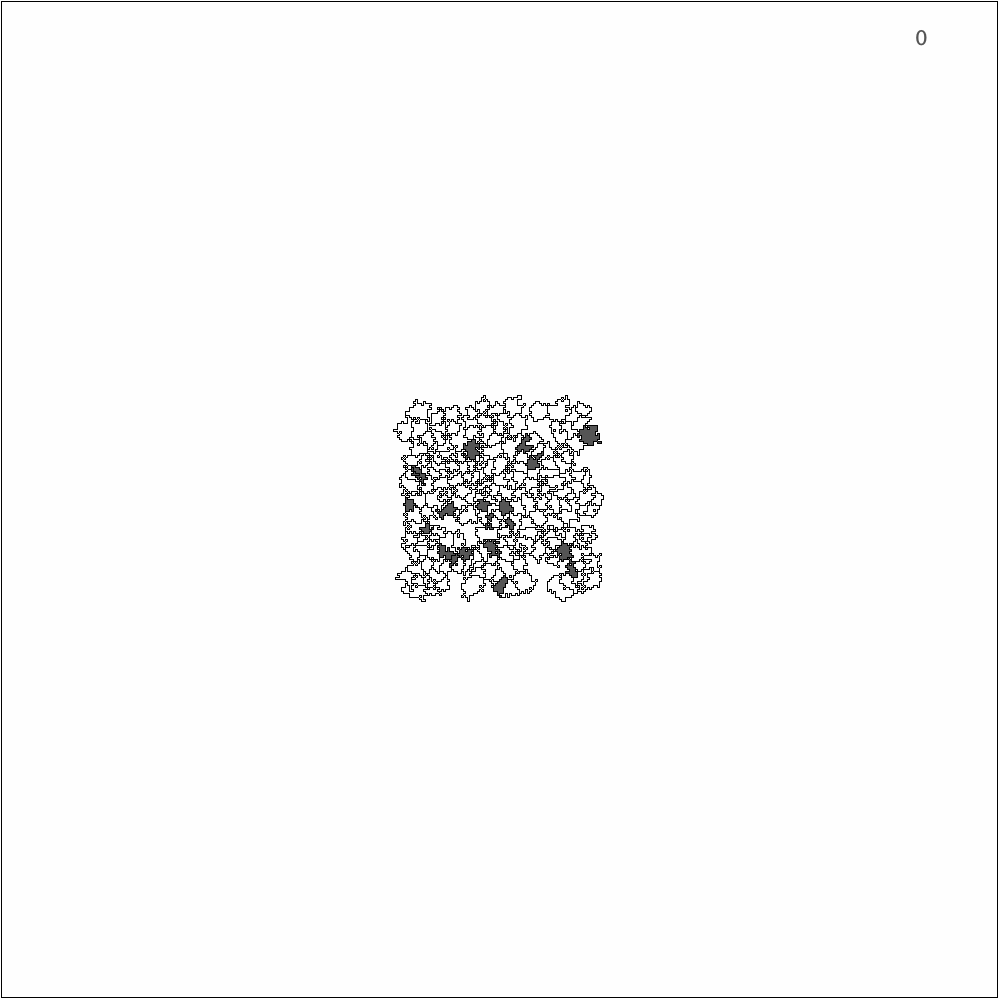

Supplement: S3 Video — See Fig 5 for details. (GIF) [file pcbi.1011825.s014.gif]
